# Renalase and its receptor, PMCA4b, are expressed in the placenta throughout the human gestation

**DOI:** 10.1038/s41598-022-08817-6

**Published:** 2022-03-23

**Authors:** Melinda Wang, Tatiana Silva, Jessica M. Toothaker, Blake T. McCourt, Christine Shugrue, Gary Desir, Fred Gorelick, Liza Konnikova

**Affiliations:** 1 Yale University School of Medicine, 375 Congress Ave, LSOG 405B, New Haven, CT 06519 USA; 2 Department of Immunology, University of Pittsburgh, Pittsburgh, PA 15213 USA; 3 Department of Pediatrics, Yale University, New Haven, CT 06520 USA; 4 Department of Obstetrics, Gynecology and Reproductive Sciences, Yale University, New Haven, CT 06520 USA; 5 Program in Human and Translational Immunology, Yale University, New Haven, CT 06520 USA; 6 Department of Internal Medicine, Yale University, New Haven, CT 06520 USA; 7 VA CT Medical Center, Yale University, New Haven, CT 06520 USA; 8 Department of Cell Biology, Yale University, New Haven, CT 06520 USA

**Keywords:** Developmental biology, Intrauterine growth

## Abstract

Placental function requires organized growth, transmission of nutrients, and an anti-inflammatory milieu between the maternal and fetal interface, but placental factors important for its function remain unclear. Renalase is a pro-survival, anti-inflammatory flavoprotein found to be critical in other tissues. We examined the potential role of renalase in placental development. PCR**,** bulk RNA sequencing, immunohistochemistry, and immunofluorescence for renalase and its binding partners, PMCA4b and PZP, were performed on human placental tissue from second-trimester and full-term placentas separated into decidua, placental villi and chorionic plates. Quantification of immunohistochemistry was used to localize renalase across time course from 17 weeks to term. Endogenous production of renalase was examined in placental tissue and organoids. Renalase and its receptor PMCA4b transcripts and proteins were present in all layers of the placenta. Estimated RNLS protein levels did not change with gestation in the decidual samples. However, placental villi contained more renalase immunoreactive cells in fetal than full-term placental samples. RNLS co-labeled with markers for Hofbauer cells and trophoblasts within the placental villi. Endogenous production of RNLS, PMCA4b, and PZP by trophoblasts was validated in placental organoids. Renalase is endogenously expressed throughout placental tissue and specifically within Hofbauer cells and trophoblasts, suggesting a potential role for renalase in placental development and function. Future studies should assess renalase’s role in normal and diseased human placenta.

## Introduction

At the maternal fetal interface, immune suppression and the development of an anti-inflammatory milieu is necessary to prevent maternal rejection of the haplo-allogenic fetus. Normal mammalian fetal development also requires adequate nutrient delivery and gas-exchange by the placenta. The placenta requires ordered growth and blood circulation to meet these needs. Reduced placental function can delay fetal growth, cause premature birth, and lead to fetal death^1^. The factors that mediate placental growth and function remain unclear but are critical to understand because of their pathologic importance and potential to be a therapeutic target^1^.

Renalase (RNLS), a secreted flavoprotein found in the plasma, was initially described to be produced by the kidneys, but since has been shown to be present in other tissues^2^. RNLS has two functional domains; one has pro-survival and anti-inflammatory features that protects tissues from various forms of injury^3^. This includes acute injury to the kidney, heart, and pancreas^3^. RNLS also has a separate oxidase domain that has been linked to NADPH and catecholamine metabolism^4^. The major receptor for extracellular RNLS is the plasma membrane calcium channel, PMCA4b, which effects downstream intracellular signaling^5–7^.

The changes seen in RNLS knockout mice reflect some of its homeostatic functions. Indicative of RNLS’ function in catecholamine metabolism, knockout mice have elevated blood pressure and an increased heart rate^2^. Validating the importance of RNLS in supporting an anti-inflammatory milieu, knockout mice also have elevated inflammatory cytokines at baseline such as TNF-α and IL-6^4^. Knock out mice have an enhanced response to acute injuries including experimental pancreatitis and myocardial infarction^4^. Its effects on cell growth are best demonstrated in cancer models such as pancreatic cancer and melanoma where inhibition of RNLS signaling is associated with tumor cell cycle arrest and apoptosis^5,6,8,9^. These findings demonstrate the importance of RNLS in physiologic regulation of blood pressure, response to injury, stimulation, and maintenance of cell growth. Single nucleotide polymorphisms have been described in clinical studies that are associated with disease. Interestingly, polymorphisms affecting the 5’ flanking region and exon/intron border, leading to reduced RNLS gene expression^10^, have been associated with hypertension and have a higher prevalence among women with preeclampsia, a condition during pregnancy characterized by hypertension and organ damage, including kidney disease^10–12^.

Studies have shown that maternal blood RNLS levels change over the course of pregnancy. Most have found elevated blood RNLS levels during pregnancy compared to non-pregnant counterparts and reduced blood RNLS levels among pregnant women with preeclampsia^10,12–16^.

RNLS functions as a growth and anti-inflammatory factor, and its association with preeclampsia suggest that it might directly affect placental function. This could occur by maternal or fetal delivery of RNLS to the placenta or endogenous placental production. To explore the role of RNLS in the placenta, we used immunolocalization to detect RNLS in placental tissues during various stages of pregnancy. Along with RNAseq data, our findings demonstrate that the RNLS is present and endogenously produced in the placental tissue and that placental RNLS levels vary throughout gestation. Furthermore, RNLS is concentrated in specific placental cell types such as Hofbauer cells and trophoblasts. These findings suggest that placental RNLS may have specific roles in placental homeostasis.

## Results

### RNLS and PMCA4b expression is detected in placental tissue

**Figure 1:**
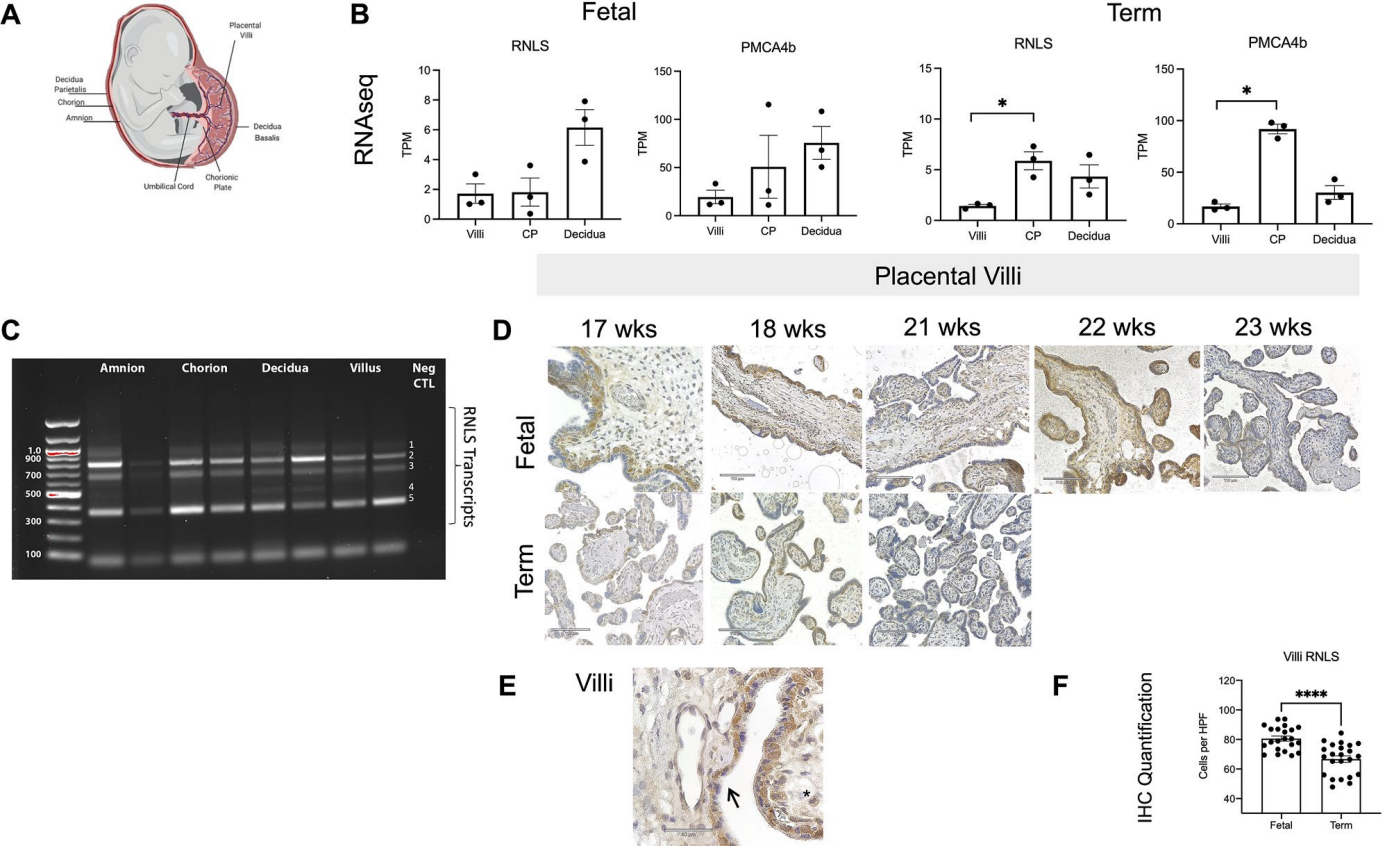
RNLS is present in placental villi throughout gestation. **(A)** Cartoon image of a fetus with labeled parts of the placenta. Made with BioRender. **(B)** Expression of RNLS and its receptor, PMCA4b, in villi, chorionic plate (CP), and decidua in fetal and term tissue (n = 3 for both). TPM- transcripts per million reads. *p-value = 0.0250 (Term RNLS); p-value = 0.0107 (Term PMCA4b) upon post hoc analysis after nonparametric Kruskal Wallis (K-W) test. Alpha level = 0.05. **(C)** PCR analysis of term placental layers performed in duplicate n = 1. **(D)** RNLS labeling in placental villi throughout development during fetal and term-time points. **(E)** Representative placental villi labeling with black arrow pointing to a region of RNLS localization along the border of placental villi and * indicating cells staining for RNLS within the villi interstitium. Immunohistochemistry with hematoxylin staining for RNLS using m28-RNLS antibody at  20x (D) and  60x ** (E)**. **(E)** Quantification of RNLS positive cells in placental villi during fetal and term-time points (n = 6). HPF-high-power field. *p-value < 0.0001 upon post hoc analysis after two-tailed Mann–Whitney test. All graphs use the mean for average values and standard error for error bars. Black arrow pointing at RNLS positive cells.

To investigate if RNLS and its receptor PMCA4b is found in the human placenta, we performed bulk RNA sequencing of three components of the placenta, maternally derived decidua and second-trimester placental villi (villi) and chorionic plate fetal membranes (CP) (Fig. 1A). RNA sequencing analysis of three-term and three second-trimester placentas demonstrated that both RNLS and PMCA4b are expressed in all three placental layers (Fig. 1B). No difference in expression was observed in second-trimester placentas, whereas RNLS and PMCA4b were enriched in CP compared to villi in the full-term placentas. To further validated this, we performed PCR analysis of RNLS transcripts from a full-term placenta (Table S1). Consistent with the bulkseq data, PCR analysis showed expression of RNLS in all tissue layers. Interestingly, multiple isoforms were expressed in the placenta with band number 4 unique to the decidual layer (Fig. 1C).

### Renalase protein expression in the placenta is present throughout human gestation

**Figure 2:**
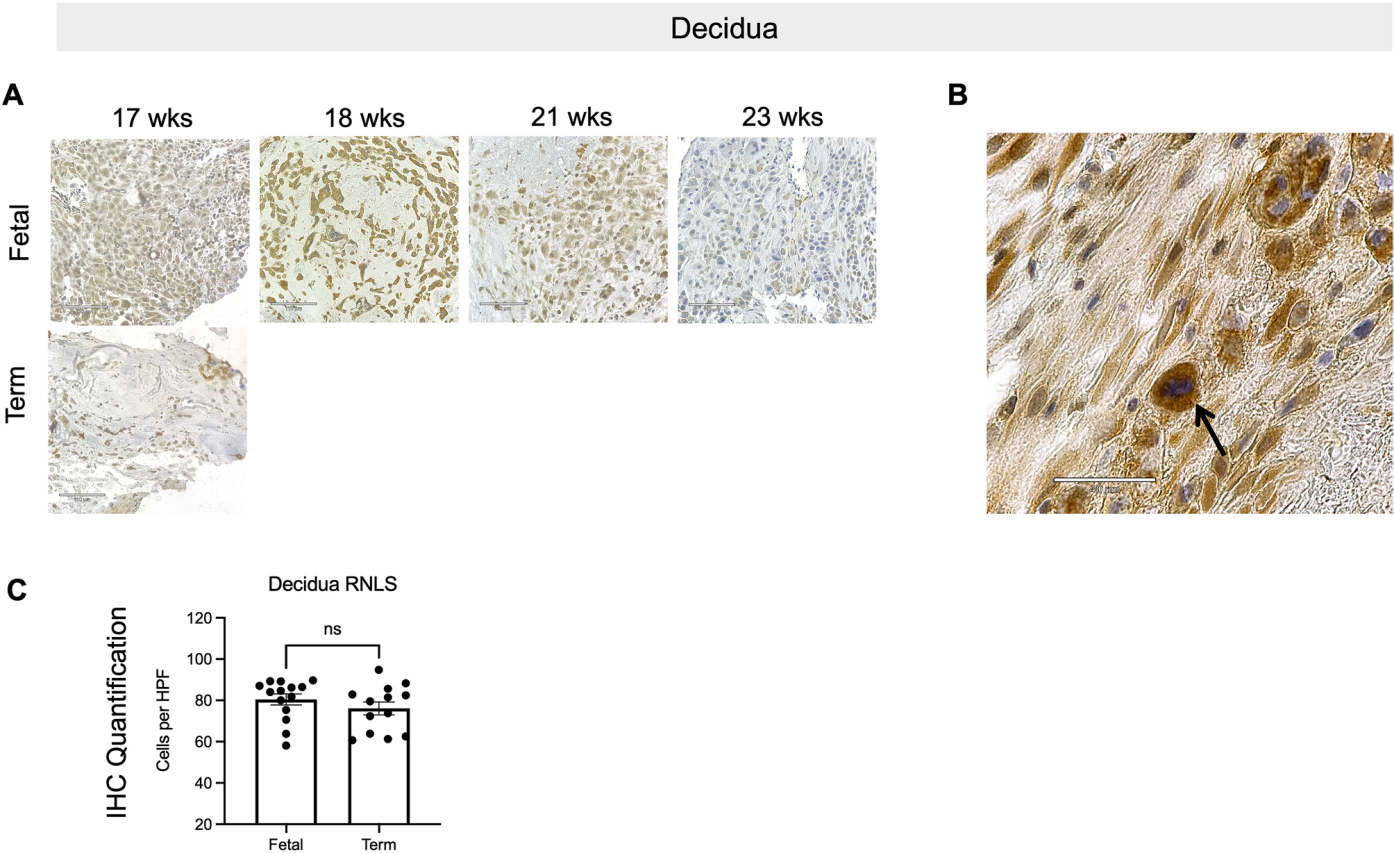
RNLS is detected in the decidua. **(A)** RNLS staining in decidua throughout development during fetal and term-time points. **(B)** Representative decidua with an arrow pointing to region of RNLS staining in a cell within the decidua. Immunohistochemistry with hematoxylin staining for RNLS using m28-RNLS antibody at  20x **(A)** and  60x **(C)**. **(C)** Quantification of RNLS staining during fetal and term-time points in the decidua (n = 4). HPF-high-power field. Post hoc analysis completed after two-tailed Mann–Whitney test. All graphs use the mean for average values and standard error for error bars. Black arrow pointing at RNLS positive cells.

**Figure 3:**
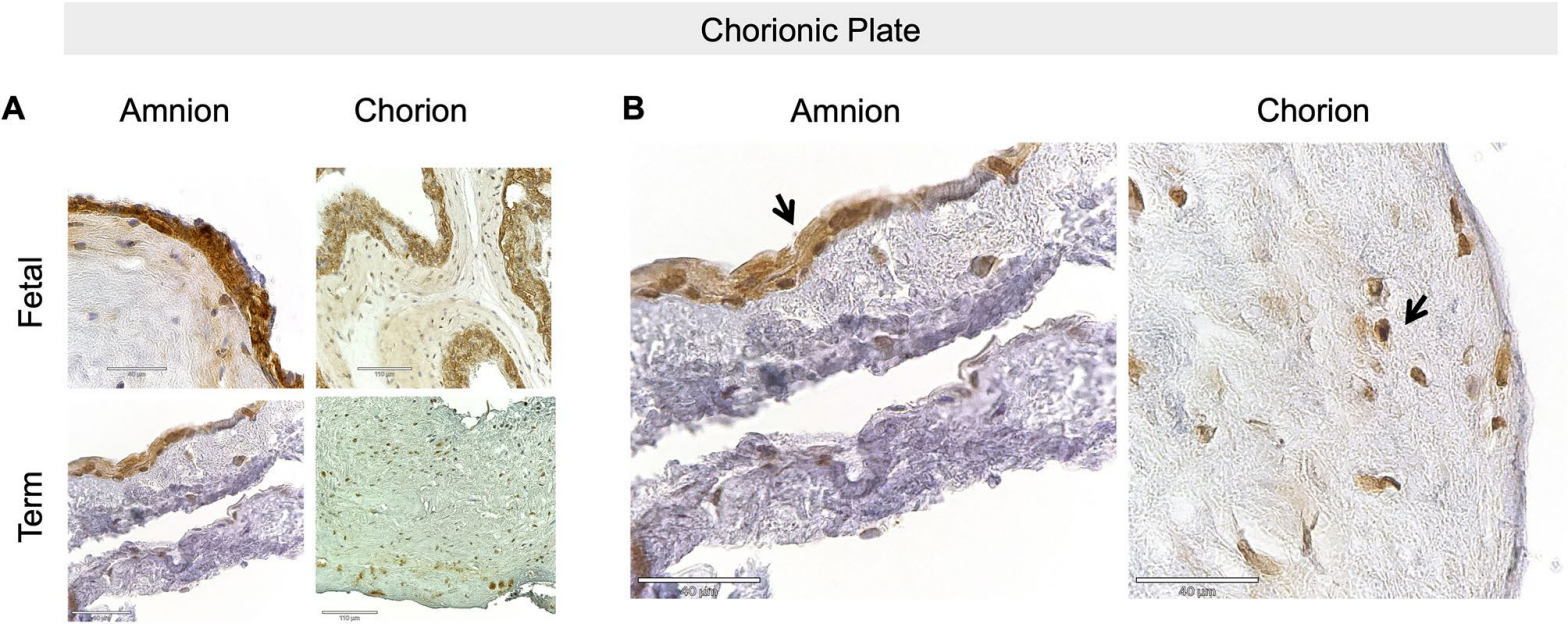
Fetal membranes contain RNLS. **(A)** RNLS labeling in amnion and chorion of the chorionic plate throughout development during fetal and term-time points. **(B)** Representative amnion and chorion RNLS staining with arrows pointing to regions of RNLS staining. Immunohistochemistry with hematoxylin staining for RNLS using m28-RNLS antibody at  20x **(A)** and 60x **(B)**. Black arrow pointing at RNLS positive cells.

To further investigate when RNLS is present during gestation in human placentas, we collected placentas from fetal cases (17–23 weeks gestational age) and term cesarean section deliveries (Table S1). RNLS protein detected by immunohistochemistry (IHC) was present throughout placental development in villi from 17 weeks to term (Fig. 1D). RNLS labeling was most intense along the outer edge of the villi. Lighter RNLS labeling was present within the interstitium of the villi, particularly around villous blood vessels and in other interstitial cells (Fig. 1D,E). Quantification of RNLS positive cells demonstrated a reduction in RNLS labeling from fetal to term cases in the villi (Fig. 1F). RNLS was similarly present within clusters of cells in the decidua from 17 weeks to term samples with no difference in the labeling between fetal and term cases (Fig. 2A–C). Within CP fetal membranes, RNLS labeling was intense in the outer layer of the amnion and within cells throughout the interstitium of the chorion in fetal and term tissues. Insufficient amount of fetal CP tissue prohibited quantification of gestationally related changes in RNLS labeling (Fig. 3A,B).

### RNLS is localized to trophoblasts and Hofbauer cells in placental tissue

**Figure 4:**
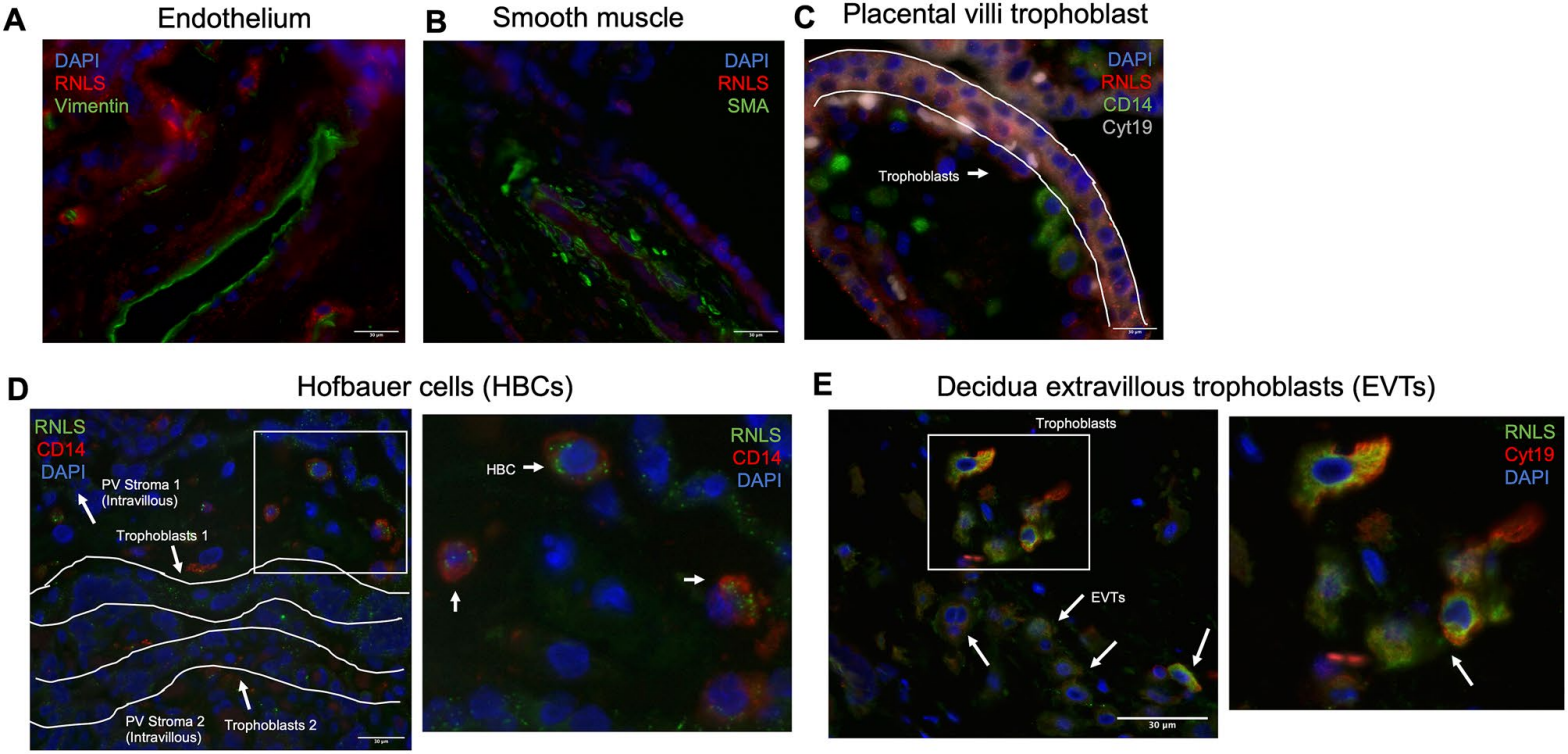
RNLS immunoreactivity is present in trophoblasts and macrophages within placental villi and decidua. **(A)** Immunofluorescence co-labeling with RNLS (red), vimentin (green, endothelium), DAPI (blue) of placental villi at  60x. **(B)** RNLS (red), SMA (green, smooth muscle actin, smooth muscle), and DAPI (blue) of placental villi at  60x. **(C)** RNLS (red), CD14 (green, Hofbauer cell), Cyt19 (white, cytokeratin 19, trophoblast), DAPI (blue) in placental villi at  60x. **(D)** RNLS (green), CD14 (red, Hofbauer cell) and DAPI (blue) of placental villi trophoblasts at  60x. **(E)** RNLS (green), Cyt19 (red, extravillous trophoblast, EVT), DAPI (blue) of placental decidua at  60x.

To determine which cells contain RNLS, immunofluorescence (IF) labeling was performed on villi, decidua, and CP. Some RNLS labeling was present around blood vessels. However, co-labeling with SMA (smooth muscle actin, a marker for smooth muscle cells that surrounds endothelium) only occasionally showed co-distribution with RNLS within the interstitium of villi (Fig. 4B). Similarly, co-labeling of RNLS with vimentin, an endothelial marker, did not demonstrate co-distribution in villi (Fig. 4A). RNLS IHC labeling was the brightest around the outside of the villi. Consistent with this, RNLS labeling co-distributed with trophoblasts, as marked by Cyt19 (cytokeratin 19, epithelial marker expressed on trophoblasts), within the villi (Fig. 4C). RNLS labeling is similarly co-distributed with Cyt19 in the decidua marking extravillous trophoblasts (EVT) (Fig. 4E). Additionally, some Hofbauer cells, as marked by CD14, also labeled with RNLS within the interstitium of the villi (Fig. 4D).

### RNLS receptor, PMCA4b, and potential binding protein, PZP, are present in placental tissue

**Figure 5:**
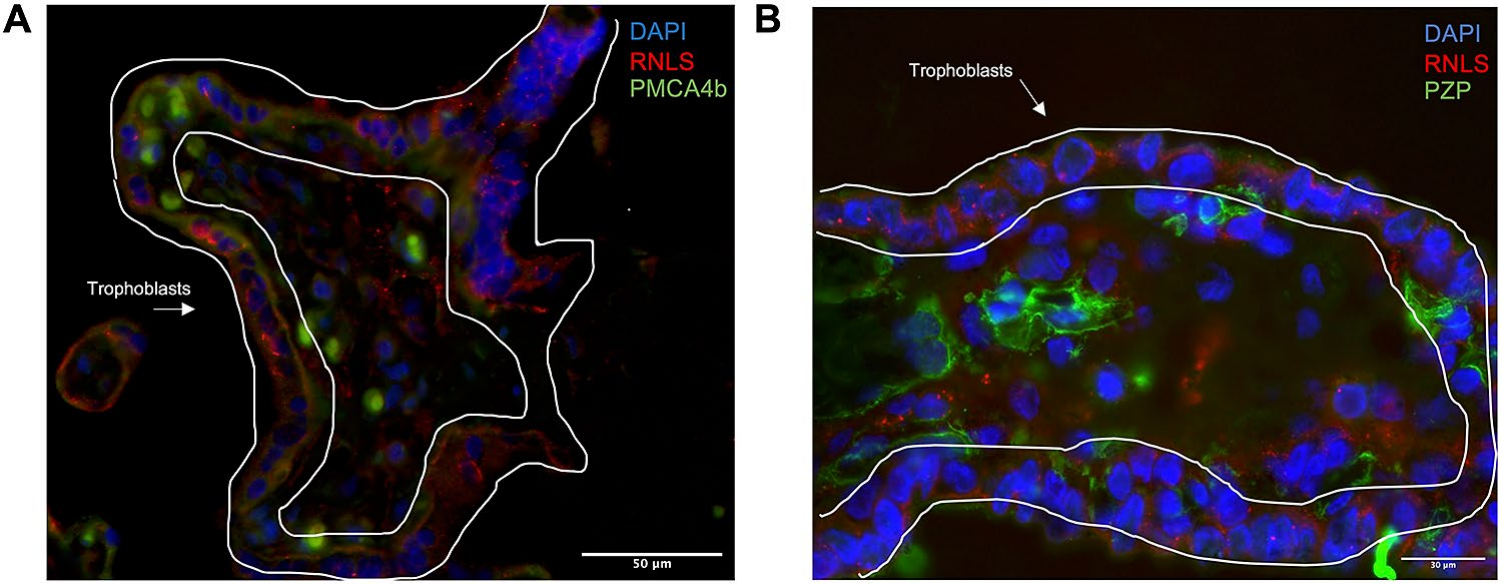
RNLS receptor and potential binding protein are present in placental villi; PMCA4b localizes to the same cells as RNLS. **(A)** RNLS (red), PMCA4b (green, RNLS receptor), and DAPI (blue) of placental villi at  60x; **(B)** RNLS (red), PZP (green, RNLS binding protein), DAPI (blue) of placental villi at  60x.

Extracellular RNLS binds to its cell-surface receptor, PMCA4b, to regulate downstream intracellular signaling^6^. Bulk RNA sequencing data demonstrated that PMCA4b is present within the placental tissue, including villi, decidua, and CP. PMCA4b and RNLS both localized to trophoblasts (Fig. 5A). However, PMCA4b localized to the basolateral aspect of syncytiotrophoblasts in contrast to RNLS, which localized mainly to the apical aspects of the syncytiotrophoblasts in villi (Fig. 5A). Cytotrophoblasts did not exhibit this polarization and RNLS and PMCA4b co-localized (Fig. 5A).

Pregnancy Zone Protein (PZP), a protease inhibitor and modulator of inflammation and immune responses, has been found in previous literature to be a candidate binding protein to RNLS within serum^17,18^. Additionally, PZP is known to increase in serum during pregnancy and accumulate at the maternal–fetal interface^19^. On immunofluorescence, PZP and RNLS did not extensively co-localize (Fig. 5B).

### Trophoblasts endogenously produce RNLS and its binding partners

**Figure 6:**
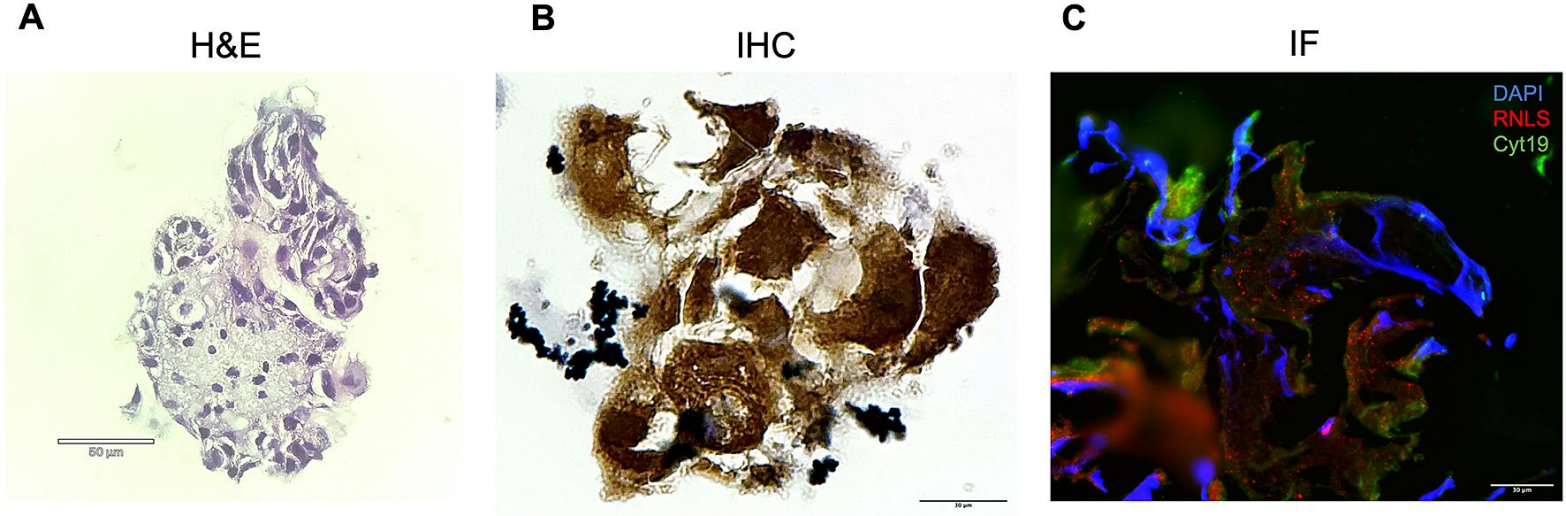
RNLS is produced endogenously in placental trophoblasts. **(A)** Hematoxylin and eosin stain of placental organoid at  40x; **(B)** immunohistochemistry staining of RNLS in placental organoid at  60x; **(C)** immunofluorescence of RNLS (red), Cyt19 (trophoblast, green), and DAPI (blue) of placental organoid at  60x.

**Figure 7:**
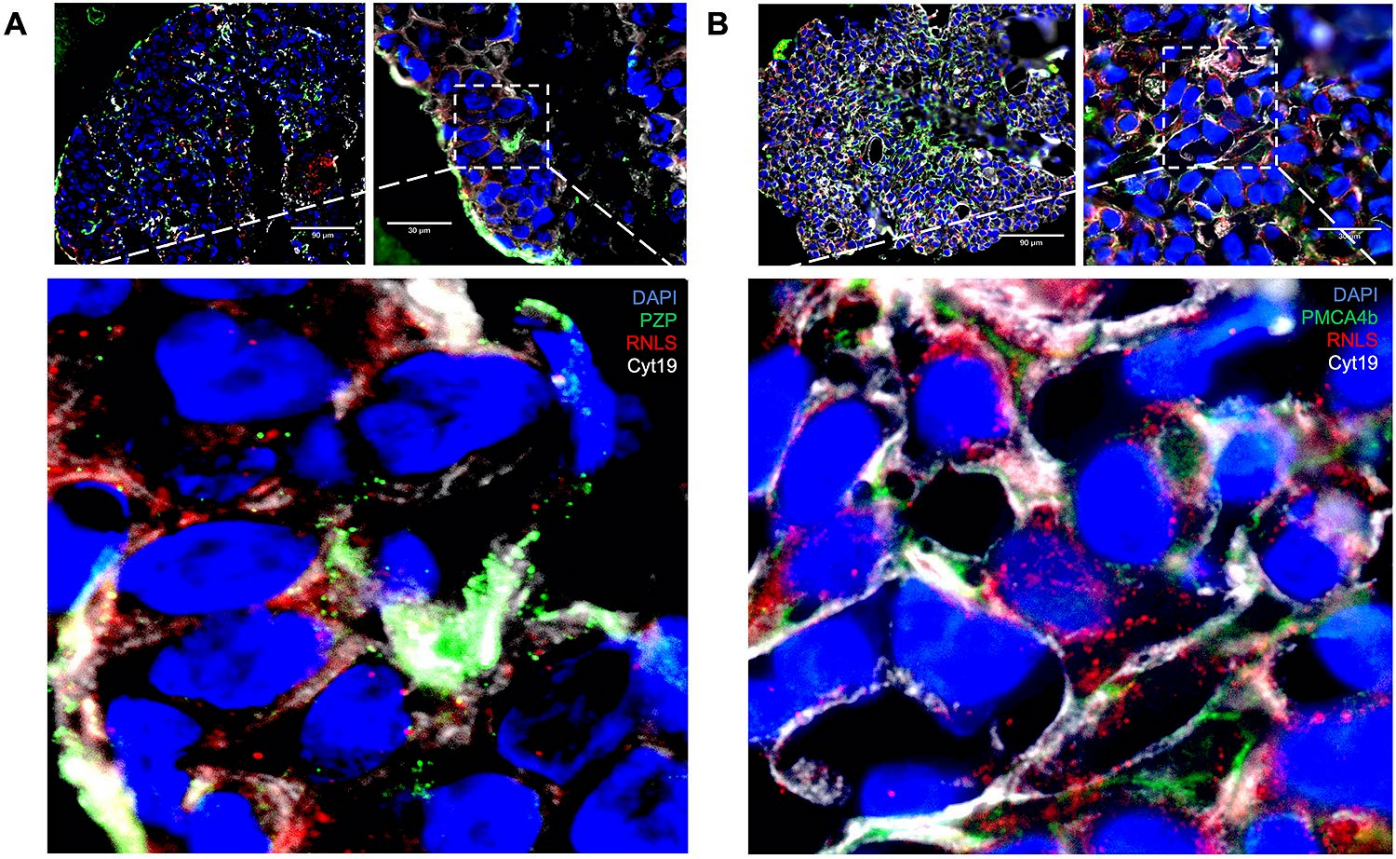
Placental villi organoids express RNLS, its receptor PMCA4b, and the RNLS carrier protein, PZP. **(A)** Immunofluorescence co-staining with RNLS (red), PZP (green), DAPI (blue), and Cyt19 (white) of placental villi organoid at  20x and  60x. **(B)** Immunofluorescence co-staining with RNLS (red), PMCA4b (green), DAPI (blue), and Cyt19 (white) at  20x and  60x.

Our RNLS sequencing data demonstrated that at least some cells within the placental villi can express RNLS. To establish whether placental trophoblasts endogenously produce RNLS, we examined whether RNLS is present in the organoids derived from placental villi trophoblasts. PV organoids were derived from second-trimester placental villi and cultured in RNLS free media (Figs. 6, 7). Both IHC and IF staining demonstrated RNLS to be abundantly present on placental trophoblasts as marked by cytokeratin (Cyt19) staining in the organoid model (Fig. 6B,C), suggesting that RNLS is synthesized endogenously by trophoblasts. Additionally, co-staining with both PMCA4b and PZP demonstrated that both the receptor and carrier protein are also endogenously produced by placental trophoblasts and can be found in proximity to RNLS (Fig. 7A,B).

## Discussion

RNLS has both anti-inflammatory and pro-survival functions that, when dysregulated, have been associated with enhanced injury and reduced cancer cell survival^3,5,6^. Preeclampsia is characterized by high blood pressure, reduced kidney function, and proteinuria, and its mechanism of action is under active investigation^13^. RNLS deficiency is associated with end-stage renal disease and hypertension^2,4,11^. Given the association between RNLS and hypertension, several studies have explored the association between RNLS and preeclampsia in pregnancy and found that lower RNLS levels correlated with preeclampsia. Although alterations in RNLS serum levels have been associated with preeclampsia, to our knowledge, no studies have explored whether RNLS can be found in placental tissue and associated with human placental development. In mice, RNLS expression is increased in reproductive and steroidogenic systems during pregnancy, suggesting a potential association between RNLS and hormonal and reproductive regulation^20^. Additionally, the invasive, proliferative, and migratory characteristics of placental cells are often compared to similar properties of cancer cells^21^. Like cancer cells, placental cells are highly proliferative, lack cell-contact inhibition, and escape the immune system, especially during the first trimester, in normal placental development^21^. Additionally, the maternal–fetal interface or the placenta is an interface between two organisms that are haplo-allogenic to one another and as such, active mechanisms to suppress rejection are necessary. We therefore hypothesized that RNLS expression would be present during normal placental development and involved in trophoblast survival and placental immune homeostasis. This is the first report regarding RNLS in the placenta. This study demonstrates that RNLS is endogenously produced by placental trophoblasts and is expressed throughout maternal and fetal tissue during normal pregnancy, including the placental villi, chorionic plate fetal membranes, and the placental villi decidua. RNLS co-localized with extra-villous trophoblasts within the decidua, suggesting fetal origin of RNLS production.

Trophoblasts are often compared to cancer cells in their similarities of escaping the immune system, proliferation, and migration^21^. After implantation, the trophoblasts invade through the primary syncytium in the villous developmental stage. Additionally, individual cytotrophoblasts invade the decidua to become extravillous trophoblasts. Trophoblasts directly contact maternal blood and glandular secretions and act as the primary site of maternal and fetal exchange of gas and nutrients. Therefore, both apical and basal sides of the trophoblast cells therefore express multiple transporters for amino acids and glucose and receptors for growth factors and hormones. Additionally, due to their proximity to maternal circulation, trophoblasts act as an immunological barrier at the fetal-maternal interface^1^. Renalase expression is upregulated by a hypoxic-inducible factor (HIF-1a) mechanism under hypoxic conditions, which has been implicated in both ischemic heart disease and renal ischemia^22,23^. The HIF system also plays a prominent role in the developing placenta, also a hypoxic environment^24^. Therefore, it is possible that RNLS levels are increased by a HIF-1a mechanism within the placenta. Trophoblasts are also a site of catecholamine expression with localization in cytotrophoblasts during early pregnancy and in syncytiotrophoblast cells during late pregnancy^25^. Additionally, RNLS is known to metabolize catecholamines with implications in hypertension and chronic kidney disease^26^. Whether renalase’s role in catecholamine metabolism has clinical significance in placental development or fetal brain development and programming requires further evaluation^25^.

RNLS expression within trophoblasts throughout placental development in placental villi, decidua, and fetal membranes, suggests a potential role for RNLS in trophoblast function. Multiple isoforms of RNLS have been detected in various tissues, although their exact roles are unclear (reviewed in Pointer, Gorelick and Desir, 2021)^27^. Interestingly, multiple RNLS isoforms were expressed in placental tissue, with one unique to the decidua. Isoform specific functions in the placenta should be investigated further. RNLS expression is consistently present along both apical and basal aspects of the syncytiotrophoblast, but its receptor, PMCA4b, localizes to the basal side of the trophoblast. However, no polarity in RNLS or PMCA4b was observed in cytotrophoblasts, perhaps suggesting differential roles for RNLS in trophoblast subtypes. Cytotrophoblasts have increased placental metabolic activity compared to syncitiotrophoblasts, and RNLS may contribute to this difference in metabolic activity^28^. Additionally, overall RNLS labeling was higher earlier in placental development during fetal periods than at term. Whether RNLS plays an immunological or pro-survival and growth factor-like role in placental development as it does in cancer cell survival requires further evaluation^5^.

Hofbauer cells (HBC) are resident macrophages (Mϕ) in the placenta that play a role in protection against vertical infection, placental vascular and trophoblast development, and in nutrient transfer^1^. HBC are classically thought of as M2-like or anti-inflammatory Mϕ. RNLS has an anti-inflammatory function and is expressed in tumor-associated macrophages in melanoma^3,6^. Additionally, dysregulated RNLS signaling in tumors can lead to macrophage polarization towards a M2-like phenotype^3^. In the current study, RNLS was present in HBC in the placental villi Mϕ. Given the anti-inflammatory role of RNLS in Mϕ, RNLS may have a similar role within trophoblasts and HBC.

Although some RNLS labeling was present around villous blood vessels, the labeling did not co-localize to either endothelial cells or the surrounding smooth muscle cells, suggesting that RNLS may be present in a different cell type or may localize to the extracellular matrix.

Additionally, the RNLS receptor, PMCA4b, and candidate serum binding protein, PZP, were present along with RNLS within placental villi and placental organoids. Together the findings suggest that RNLS may have roles in normal placental development. Future studies should assess the function of RNLS in normal placental development and its potential role in placental pathology.

Previous studies have shown that PZP, present in maternal serum during pregnancy at elevated levels, flows into the intervillous space and not within the interstitium of the villi^19^. PZP expression has also been detected within villous mesenchymal cells, trophoblasts, and some decidual cells^19^. Additionally, PZP is a potential candidate as a binding protein to RNLS in blood^17^. In this study, we found PZP expression particularly strong along the endothelium and within the interstitium of placental villi. This potentially suggests that PZP could be required to transfer RNLS from fetal blood to placental interstitium.

Preeclampsia is associated with symptoms of hypertension and proteinuria, and reduced RNLS levels are associated with hypertension and kidney disease^3,29^. Previous studies have found an association between reduced serum RNLS levels and pregnant women with preeclampsia^12–16^. Additionally, reduced PZP levels have been associated with preeclampsia^18^. Although the mechanisms of preeclampsia remain unclear, increased inflammation has been implicated as a contributory factor. Studies suggest that trophoblasts in patients with preeclampsia may not transform from a proliferative to invasive phenotype, leading to incomplete remodeling of maternal spiral arteries, shallow placentation, and placental ischemia. Abnormal placentation may also lead to hypoxia, oxidative stress, downstream inflammation, endothelial dysfunction, and maternal systemic disease^29^. Whether RNLS plays a role in the pathogenesis of preeclampsia requires further evaluation.

Although this study is descriptive, it is the first to characterize RNLS expression within normal placental development. Given the function of RNLS in other organs and cell types, it is exciting to postulate that RNLS may have a similar role in placental biology. Future studies should further assess the function of RNLS within the normal placenta, which may elucidate its potential role in pathological placental development.

## Methods

### Placental tissue procurement

Second-trimester placental tissue was obtained through the University of Pittsburgh Biospecimen core after IRB approval by University of Pittsburgh (IRB# PRO18010491) (Table S1). Term placentas were collected through the Yale University YURS Biobank from C-section deliveries without obstetric complications after IRB approval by Yale University(IRB #2000028847) (Table S1). Tissue was collected only from subjects who signed an informed consent to allow use for research purposes. All samples were deidentified.

### Placental tissue processing

Placental villi were separated using forceps under a light dissection microscope (Fisherbrand #420430PHF10) from the chorionic and amniotic membranes lining the chorionic plate (CP) and from the decidua basilis (referred to as decidua throughout the manuscript) on the basal plate side of the placenta. Tissue was thoroughly washed with PBS prior to cryopreservation, snap freezing, and placement in formalin before embedding in paraffin. Each layer of tissue was preserved separately.

### RNA sequencing

Snap frozen placental tissues were shipped on dry ice to MedGenome for mRNA extraction and library preparation. RNA extractions were completed with the Qiagen All Prep Kit (#80204). cDNA synthesis was prepped with the Takara SMART-seq kit (#634894) and NexteraXT (FC-131-1024, Illumina) was used to fragment and add sequencing adaptors. Quality control was completed by MedGenome using Qubit Fluorometric Quantitation and TapeStation BioAnalyzer. Libraries were sequenced on the NovaSeq6000 for Paired-End 150 base pairs for 90 million reads per sample.

### RNA sequencing analysis

FASTQ files were imported and subsequently analyzed with CLC Genomics Workbench 20.0 (https://digitalinsights.qiagen.com). Briefly, paired reads were first trimmed with a quality limit of 0.05, ambiguous limit of 2 with automated read through adapter trimming from the 3’-end with a maximum length of 150. Trimmed reads were then mapped to the homo_sapiens_sequence_hg38 reference sequence. TPM values were extracted and used as reference for gene expression values.

### qPCR

To prepare complementary DNA (cDNA), snap frozen samples of amnion, villi, chorion, and decidua were each placed in individual RNase-free tubes with TRIzol™ reagent (Thermo 15596018) and homogenized. Chloroform (Thermo 203843) was added to the samples, which were then centrifuged and separated by an aqueous and sediment layer. Isopropanol (ACS 2624025) was added to the aqueous later and centrifuged to generate an RNA pellet, which was resuspended in 75% ethanol and subsequently air dried. Pellets were then resuspended in RNase-free water and stored at −80 °C overnight. cDNA was synthesized using the LunaScript^®^ RT SuperMix Kit (E3010).

PCR was carried out using 1.5 µL first-strand cDNA in a 50 µL reaction volume containing 1X PCR Buffer, 1.5 mM MgCl_2_, 200 µM of each deoxynucleotide triphosphate, 2 units Platinum *Taq* DNA polymerase (Invitrogen, Carlsbad, CA) and 200 nM each of primers 1 + (5’-ATGGCGCAGGTGCTGATCG-3’) and 1029- (5’-CTAAATATAATTCTTTAAAGCTTCCAGAACACATAGGGC-3’). Amplification conditions were an initial denaturation for 2 min at 94 °C then 40 cycles of denaturation (94 °C, 30 s), annealing (55 °C, 30 s), and extension (68 °C, 60 s). This was followed by a single extension step for 10 min at 72 °C. PCR products were analyzed on agarose gels that contained ethidium bromide.

### Immunohistochemistry

Immunohistochemistry procedure was performed as previously described^5^. Briefly, paraffin embedded human placental tissue samples were sectioned, deparaffinized, and hydrated with Histoclear and ethanol (Sigma, St. Louis, MO, USA). Slides then underwent antigen retrieval using 10 mM sodium citrate (pH 6) buffer in a pressure cooker. Sections were blocked using DAKO Dual Endogenous Enzyme Block for Autostainer and 2.5% normal horse serum (Agilent, Santa Clara, CA, USA). Slides were then incubated with primary antibody m28-RNLS (1:500) overnight at 4 °C and secondary antibody IMPRESS Reagent anti-Rabbit IgG (Vector Laboratories, Burlingame, CA, USA). Antibody binding was detected using Vector DAB substrate kit and counterstained with hematoxylin (Vector Laboratories, Burlingame, CA, USA). Images were obtained at 20× and 60× using Echo^®^ Revolve microscope. Staining specificity was determined by labeling with secondary antibody.

### Image quantification

IHC images were deconvoluted in ImageJ/Fiji. DAB signals were quantified by providing the average pixel intensity of each IHC image (mean).

### Immunofluorescence

As described above, paraffin embedded human placental tissue samples underwent sectioning, deparaffinized, hydration, and antigen retrieval. Sections were blocked using 5% goat serum in 0.3% Triton X-100 in TBS and incubated with primary antibodies (m28-RNLS, 1:500; PZP, 1:1500, Santa Cruz Biotechnology sc12472; vimentin, 1:200, Invitrogen OMA1-06001; SMA, 1:400, Abcam ab5694; Cyt19, 1:1000, Abcam ab192751; CD14, 1:500, Abcam ab181470; PMCA4b, 1:200, Santa Cruz Biotechnology sc20027). Sections were then incubated with appropriate secondary antibody (1:1000, Invitrogen) and quenched for 2 min using TrueView autofluorescence quenching kit (Vector Laboratories, Burlingame, CA, USA). Images were obtained at 20× and 60× using Echo^®^ Revolve microscope.

### Placental organoid culture

The placental organoid culture protocol used was adapted from Turco et al. 2018^30^. The gestational age of the placenta used in organoid culture was 23 weeks (Table S1). Cryopreserved villi samples were thawed and placed into Advanced DMEM/F-12 (Dulbecco’s Modified Eagle Medium/Ham’s F-12) (Gibco, 12634-010) and washed in sterile PBS. Tissue was resuspended in fetal bovine serum (FBS) (Corning, 35-010-CV), EDTA (Invitrogen, 15575-038), Trypsin–EDTA (Gibco, 2520056) and collagenase. Tissue dissociation and single cell suspension was completed using gentleMACS™ Octo Dissociator with Heater (Miltenyi Biotec #130-096-427). Resultant cells were resuspended in Matrigel (Corning, 356234) and plated into wells with media. Placental organoid media consisted of Advanced DMEM/F-12; N2 supplement (Life Technologies, 17502048); B27 supplement minus vitamin A (Life Technologies, 12587010); Primocin (Invivogen, ant-pm-1); l-glutamine (GlutaMAX) (Life Technologies, 35050061); *N*-Acetyl-l-cysteine (Sigma, A9165-5G); ALK-4, -5, -7 inhibitor, A83-01 (Tocris, 2939); CHIR99021 (Tocris, 4423); Recombinant Human EGF (Peprotech, AF-100-15); Recombinant Human R-spondin 1 (BioTechne, 4645-RS); Recombinant Human FGF2 (Peprotech, 100-18C); Recombinant Human HGF (Peprotech, 100-39); Y-27632 (Millipore, 688000); PGE2 (Sigma, P0409); HEPES (Gibco, 15630080). Generation of placental organoids was accomplished by placing cultures in a humidified incubator at 37 °C with 5% CO_2_. Media was changed every two to three days. Organoid clusters became apparent after seven days. Quality control measures for organoid culture included staining for H&E and trophoblast markers. Cytokeratin-19 was expressed on all trophoblasts. The inside out nature of organoids was confirmed by E. cadherin labeling for cytotrophoblasts.

## Supplementary Information

Supplementary Information.

## Data Availability

RNA sequencing data is available through searching GSE184830 at https://www.ncbi.nlm.nih.gov/geo.
