# Supplementary material for: Renalase and its receptor, PMCA4b, are expressed in the placenta throughout the human gestation
Source: Sci Rep. 2022 Mar 23;12:4953. doi: 10.1038/s41598-022-08817-6 (PMC8943056; doi:10.1038/s41598-022-08817-6)
Supplement: Supplementary file 1 — Supplementary Information. [file 41598_2022_8817_MOESM1_ESM.docx]

**SUPPLEMENTARY TABLE AND FIGURE LEGENDS**

**TABLE S1. Sample’s Demographics.**


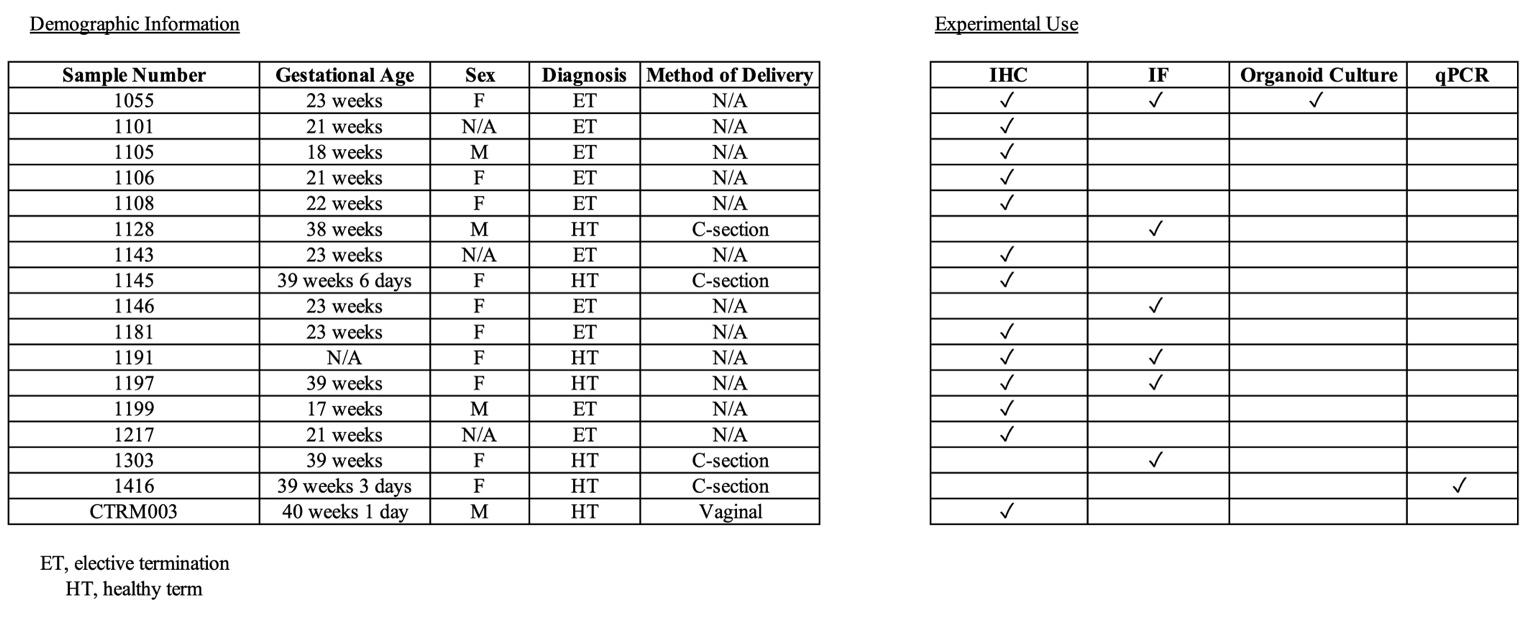


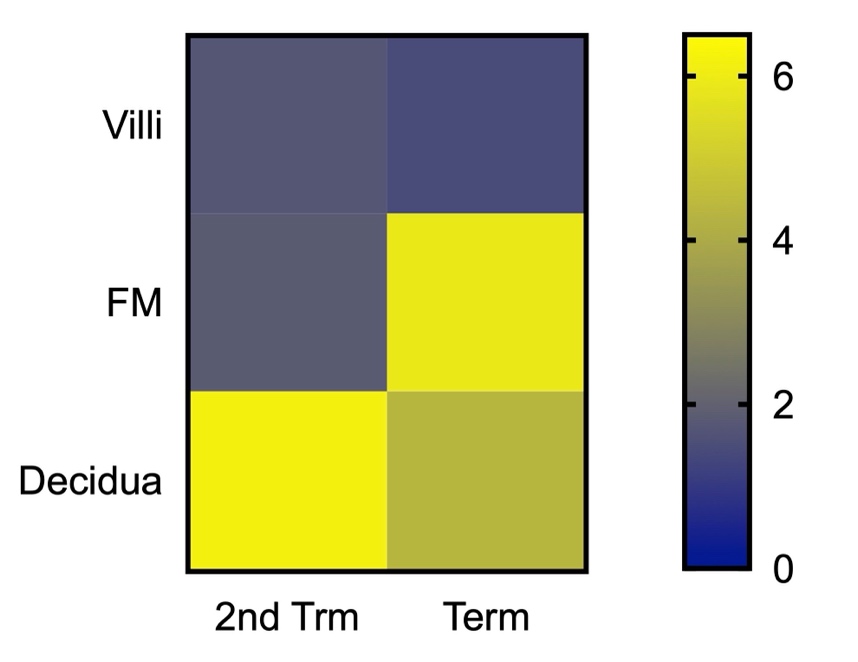


**Figure S1.** Heatmap of RNLS expression corresponding to RNA sequencing data in **Figure 1B.**


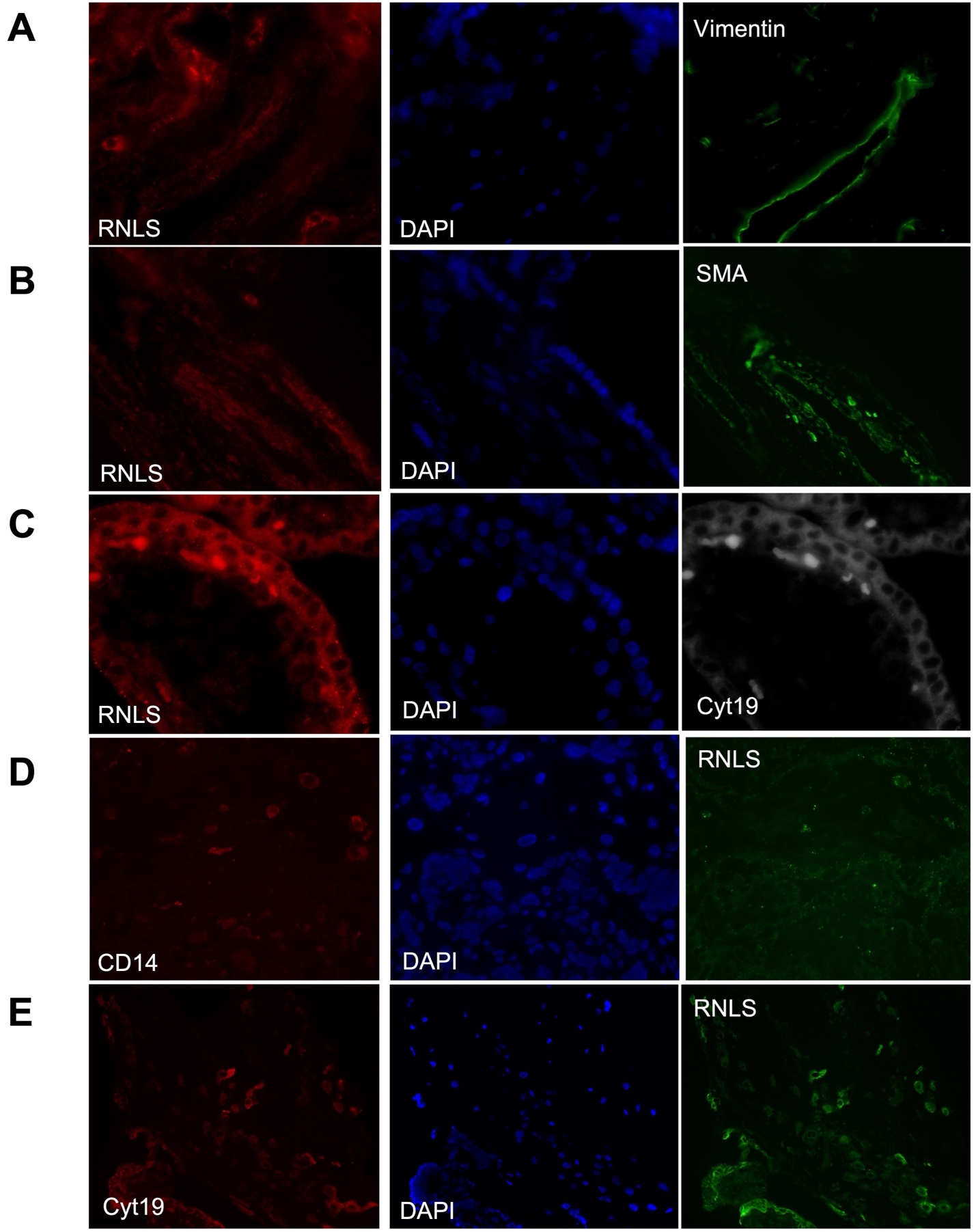


**Figure S2.** A-E. Individual channel and merged images corresponding to **Figure 4.**


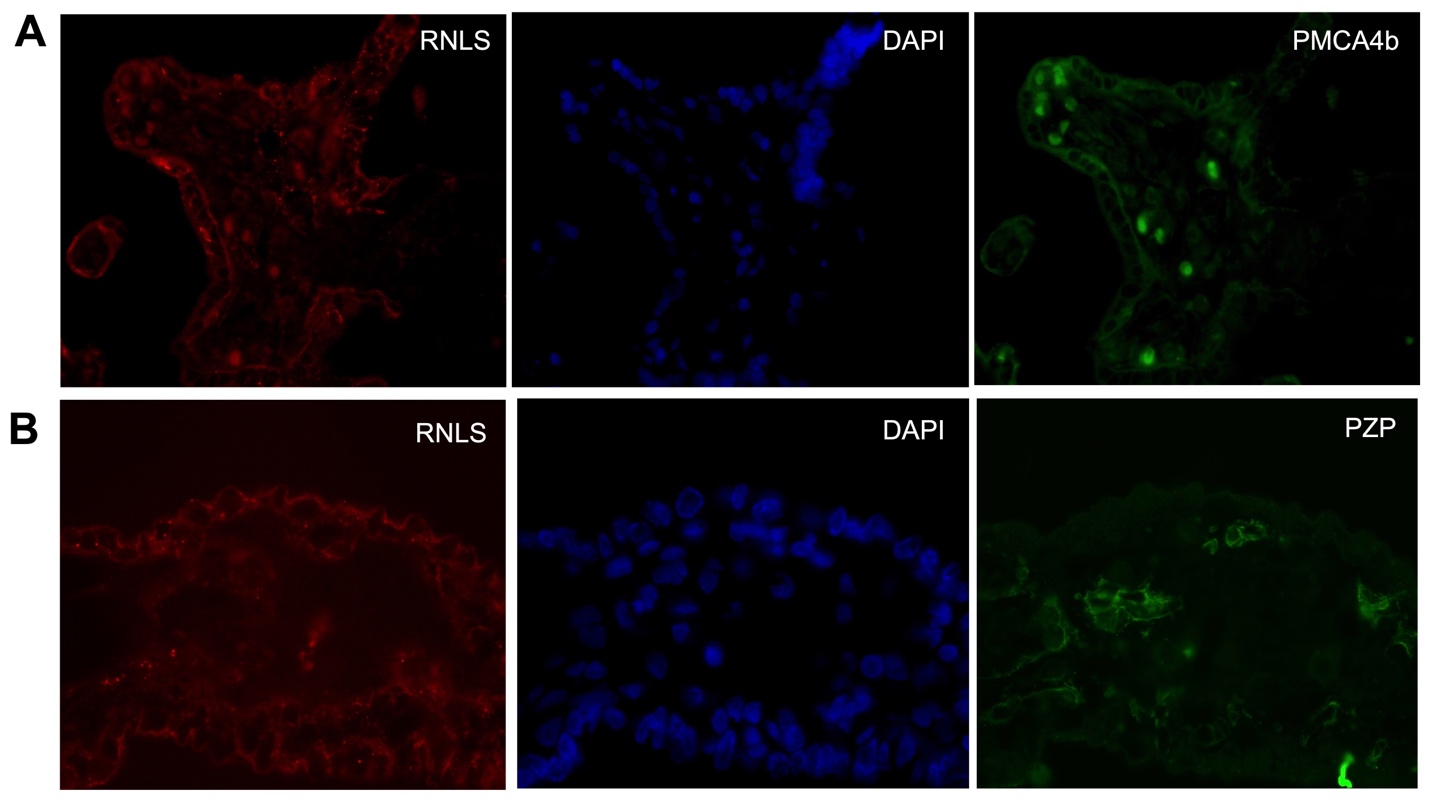


**Figure S3.** A-B Individual channel and merged images corresponding to **Figure 5**.


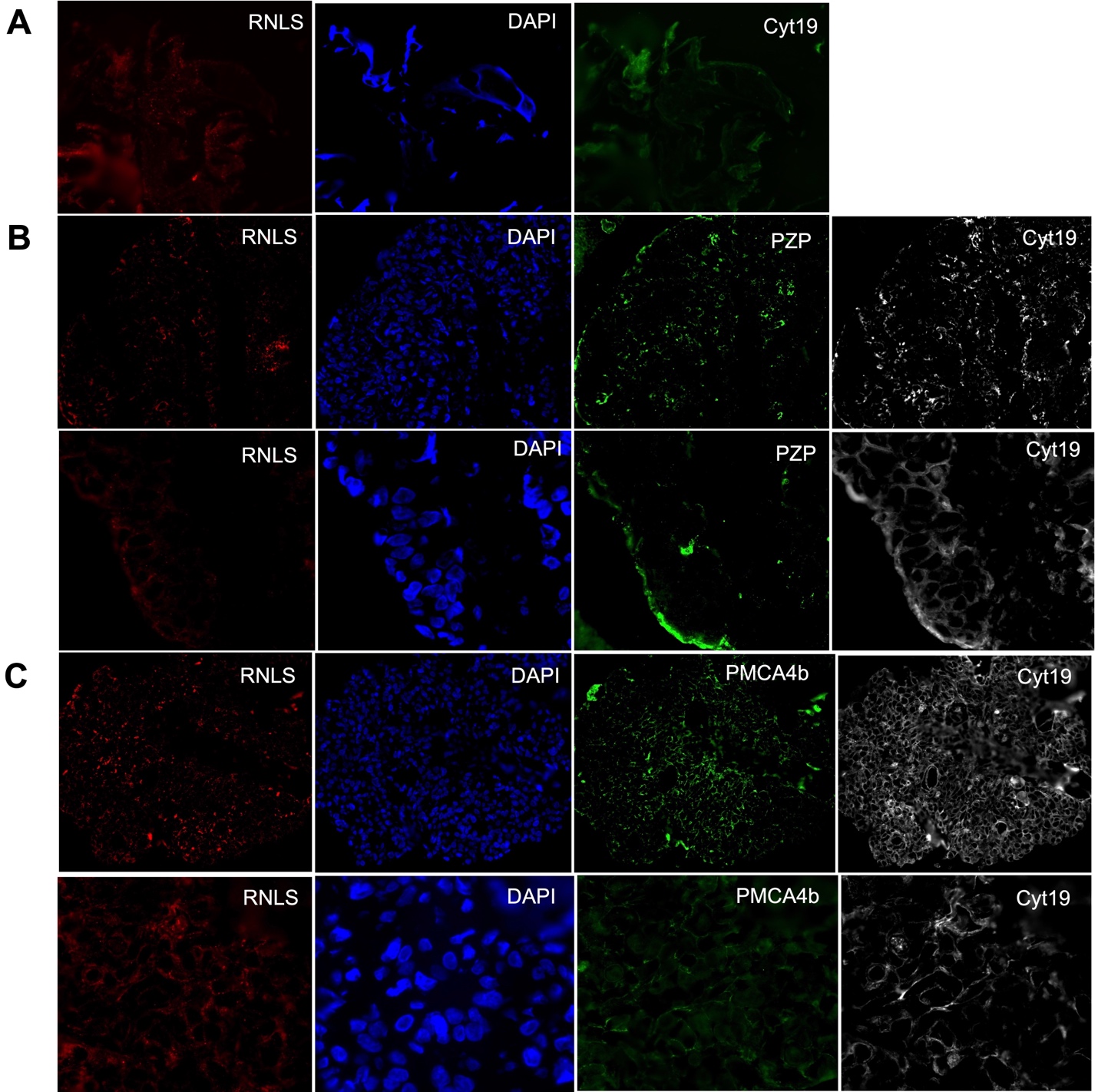


**Figure S4.** A. Individual and merged images corresponding to Figure 6. B-C Individual and merged images corresponding to **Figure 7.**
